# Anemia of Chronic Disease in Patients With Cardiovascular Disease

**DOI:** 10.3389/fcvm.2021.666638

**Published:** 2021-08-12

**Authors:** Lukas Lanser, Dietmar Fuchs, Hubert Scharnagl, Tanja Grammer, Marcus E. Kleber, Winfried März, Günter Weiss, Katharina Kurz

**Affiliations:** ^1^Department of Internal Medicine II, Innsbruck Medical University, Innsbruck, Austria; ^2^Institute of Biological Chemistry, Biocenter, Innsbruck Medical University, Innsbruck, Austria; ^3^Clinical Institute of Medical and Chemical Laboratory Diagnostics, Graz Medical University, Graz, Austria; ^4^Medical CIinic V, Medical Faculty Mannheim, University of Heidelberg, Mannheim, Germany; ^5^SYNLAB Academy, SYNLAB Holding Deutschland GmbH, Augsburg and Mannheim, Germany

**Keywords:** anemia, anemia of chronic disease, iron deficiency, immune activation, cardiovascular disease, outcome

## Abstract

**Objective:** Anemia is often found in patients with coronary artery disease (CAD) or acute coronary syndrome (ACS) and related to disease severity. Our study investigated the relationship between anemia, iron homeostasis and inflammation in CAD and examined their influence on the outcome of patients.

**Patients and Methods:** Markers of immune activation (neopterin, interleukin [IL]-12, IL-6, high sensitive C-reactive protein (hsCRP), fibrinogen, serum amyloid A [SAA]) and iron metabolism (ferritin, transferrin saturation, hemoglobin) were determined in 2,082 patients (68.7 % men, median age 63 years) from the Ludwigshafen Risk and cardiovascular Health (LURIC) cohort. Patients were followed-up for a median of 9.81 years.

**Results:** 960 patients (46.1 %) presented with chronic CAD, 645 patients (31.0 %) had an ACS, and 477 patients (22.9 %) presented with no CAD in coronary angiography (CAG). Anemia (*n* = 357, 17.1 %) was associated with disease severity (reflected by more progressed stenosis in CAG, CCS, and NYHA classes, and a lower LV-EF), a higher cardio-cerebrovascular event rate and higher levels of inflammatory markers. Interestingly, anemia was only predictive for an adverse outcome in patients with elevated inflammatory markers. Accordingly, anemia of chronic disease (ACD) was associated with a higher cardio-cerebrovascular event-rate in the subsequent 2 years as compared to patients with other types of anemia or without anemia (14.3 vs. 6.1 vs. 4.0%, *p* < 0.001).

**Conclusions:** This study confirms that anemia and immune activation are strongly related to cardiovascular disease progression and an adverse outcome. Our data suggest that the association of anemia with disease severity and outcome might mainly be due to underlying inflammation.

## Introduction

Anemia and iron deficiency (ID) are frequently encountered in patients with coronary artery disease (CAD) and acute coronary syndrome (ACS) and are both related to a poor prognosis (1, 2), a reduced functional capacity and a reduced health-related quality of life of patients (3, 4). Anemia can be the consequence of absolute ID, which is mostly due to chronic blood loss, causing iron deficiency anemia (IDA). However, in many patients enhanced formation of pro-inflammatory cytokines including interleukin 1 (IL-1), tumor necrosis factor alpha (TNF-α) or interferon gamma (IFN-γ) leads to the development of functional iron deficiency (ID) and anemia of chronic disease (ACD) (5). These pro-inflammatory cytokines suppress renal erythropoietin production, but also directly inhibit erythropoiesis in the bone marrow (6–8). In addition, cytokines like interleukin 6 (IL-6) or interleukin 10 (IL-10) and crucially also the iron master regulator hepcidin increase iron uptake and inhibit iron export from macrophages (5). Iron restriction within macrophages limits iron availability for erythropoiesis finally leading to anemia (9).

IFN-γ, which orchestrates T-helper cell type 1 (Th1) immune responses, has diverse effects on erythropoiesis and the progression of atherosclerosis. IFN-γ increases iron uptake and decreases iron release into macrophages thus reducing iron availability for erythropoiesis (10). Moreover, IFN-γ inhibits the proliferation and life span of erythroid progenitor cells (6, 11), the production of erythropoietin (12) and its receptors on erythroid progenitor cells (13). While changes in iron homeostasis occur immediately (10, 14), inhibition of erythropoiesis and consequently anemia occur only after chronic exposure to IFN-γ (11). IFN-γ further enhances immune effector pathways and biochemical pathways in human monocytes/macrophages thus enforcing the progression of atherosclerosis: IFN-γ enforces the formation of reactive oxygen species, promotes antigen presentation (15) and stimulates the production of neopterin in macrophages (16). Previous studies have already described a significant correlation between increased neopterin concentrations and anemia (17–20). Finally, higher neopterin concentrations have also been related with a poor prognosis in patients with cardiovascular disease (21–24).

As both, immune activation and anemia appear to be linked with the outcome of patients with cardiovascular disease, we wanted to investigate whether patients with ACD or multifactorial anemia differed regarding their risks for further cardiovascular events. Therefore, the objective of this study was to specify the cause of anemia in patients with CAD and ACS more precisely. Furthermore, we wanted to investigate whether the underlying cause of anemia (inflammation, ID or multifactorial anemia) has an impact on the prognosis of patients.

## Materials and Methods

### Study Population

**Figure 1:**
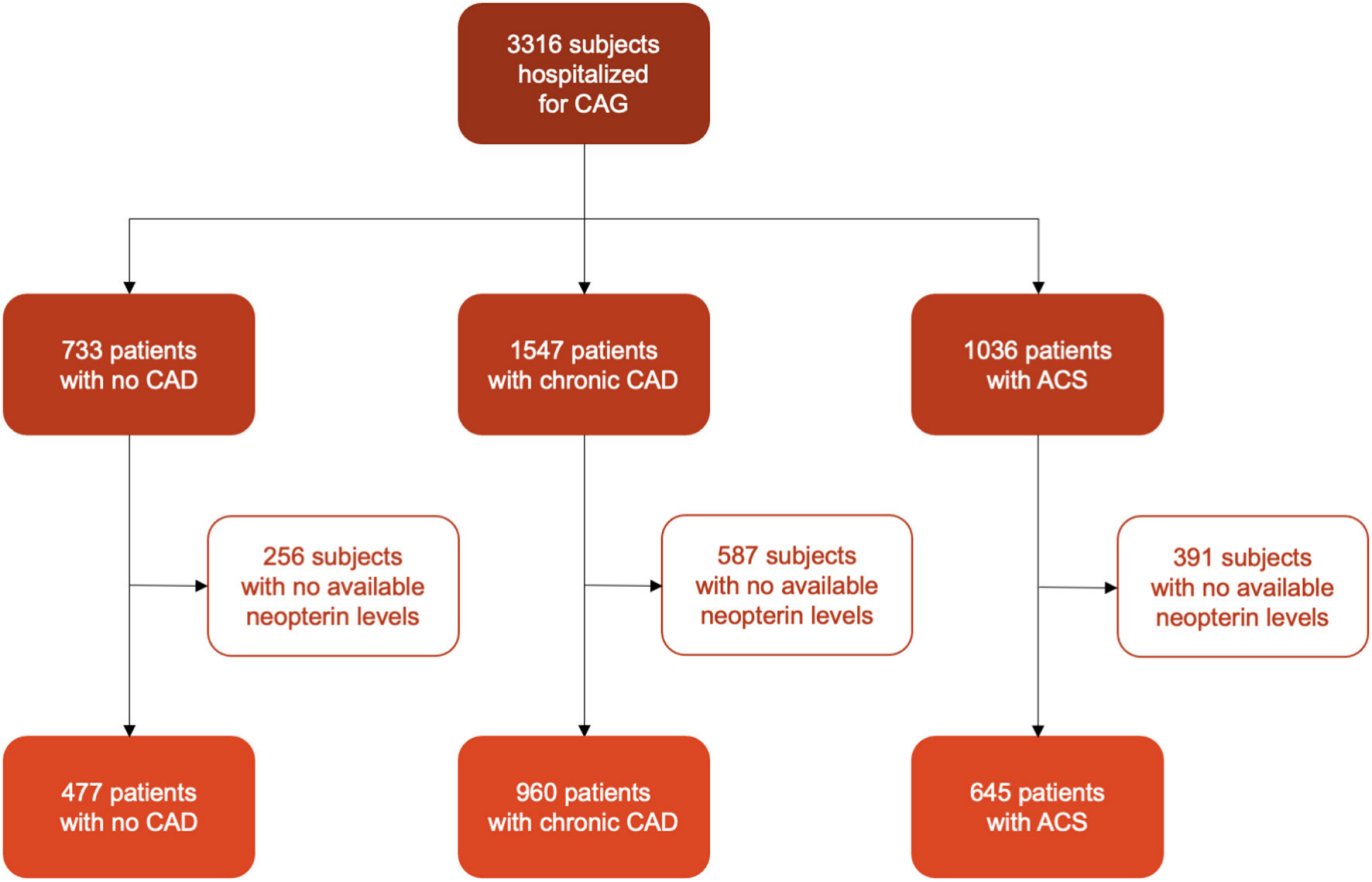
Flowchart of patients' selection.

We analyzed data of 3,316 subjects from Ludwigshafen Risk and Cardiovascular Health (LURIC) study. Subjects were hospitalized for coronary arteriography (CAG) between June 1997 and January 2000 (25) because of chest pain and/or non-invasive test results consistent with myocardial ischemia. Additional inclusion criteria were available CAG, German ancestry and clinical stability except for acute coronary syndrome (ACS). Exclusion criteria were defined as any acute illness (other than ACS), any chronic non-cardiac diseases, or malignancy within the last 5 years. For further analysis, we included 2,082 patients with available neopterin concentrations. (Flow-chart of patients selection see Figure 1).

The study conformed to the principles outlined in the Declaration of Helsiniki and was approved by the ethics committee at the Landesärztekammer Rheinland-Pfalz. All patients gave written informed consent to participate in this study.

### Angiography

Five experienced angiographers analyzed the angiograms and estimated maximal luminal narrowing by visual analysis. The three major coronary arteries were divided into 15 coronary arterial segments (26). Subjects were defined to have a clinically relevant CAD, if they presented with at least one stenosis (visible luminal narrowing) of ≥20 % in at least one of 15 coronary segments.

### Outcome Analysis

Patients were followed up over a 10-year-period. The event-free survival was defined as the period of time between the first hospitalization and patients' death. Information about patients' vital status was obtained from local community registries. Death certificates were reviewed to classify deceased patients according to whether they died from cardio-cerebrovascular or non-cardio-cerebrovascular events. Death from cardio-cerebrovascular events included sudden cardiac death (SCD), fatal myocardial infarction (MI), congestive heart failure (CHF), death after intervention to treat CAD, fatal stroke or other death due to coronary heart disease. The classification was performed by two experienced physicians who were given the death certificates without any other data of the study participants. In case of a disagreement in classification of cause of death, the final decision was made by one of the principal investigators of the LURIC study after discussion.

### Measurements

Laboratory measurements were performed at the baseline visit before CAG was performed (27). In addition, blood samples were stored at −80°C for later specific analyzes. Standard laboratory methods have been described elsewhere (25).

Hemoglobin concentrations (*n* = 2,082) were detected with the cyanmethemoglobin method (Technicon H1, Technicon GmbH, Bad Vilbel, Germany and Advia 120 Bayer Diagnostics, Tarrytown, USA). Transferrin (*n* = 2,082) was measured with an immunoturbidimetry and serum iron (*n* = 2,082) with a colorimetric ferrozine assay on a Hitachi 7171 analyzer (Roche, Mannheim, Germany). Transferrin saturation (TSAT) was defined as followed: (serum iron/5.5)/(transferrin/100) x 3.98. Ferritin concentrations (*n* = 2,082) were measured with an electrochemiluminescence enzyme immunoassay on an Elecsys 2010 Roche autosampler (Roche, Mannheim, Germany). sTfR-Ferritin-index was calculated as quotient of sTfR concentration/log(ferritin concentrations).

Serum neopterin concentrations (*n* = 2,082) were measured with a radioimmunassay (BRAHMS Diagnostica, Hennigsdorf, Germany) with a sensitivity of 1 nmol/L neopterin and an interassay coefficient of variation ranging from 3.9 to 8.2% (28). We calculated the ratio of neopterin/eGFR since neopterin is excreted by the kidney and was shown to be higher/accumulate in patients with reduced kidney function (24, 29). The high-sensitive C-reactive protein (hsCRP; *n* = 2,082) was quantified by immunonephelometry (N High Sensitive CRP, Dade Behring, Marburg, Germany). IL-6 concentrations (*n* = 2,079) were detected by ELISA (R&D Systems Inc. USA). IL-12 concentrations (*n* = 1,809) were also measured by ELISA (BioSource Europe SA Nivelles, Belgium). Fibrinogen levels (*n* = 2,080) were measured with the Clauss method (STA fibrinogen/STA Stago, Stago Diagnostica/Roche, Mannheim, Germany). Serum amyloid A (*n* = 2,082) was measured using nephelometry (Behring Nephelometer II Analyzer).

Creatinine was measured with the method of Jaffé at 37°C with a Hitachi 717 autoanalyzer and commercial kits (Roche Diagnostics, Mannheim, Germany) (30). Estimated glomerular filtrations rate (eGFR) was defined as followed (CKD-EPI equation): eGFR (mL/min/1.73 m^2^) = 141 x min[serum creatinine (mg/dL)/κ or 1]^α^ x max[serum creatinine (mg/dL)/κ or 1]^−1, 209^ x 0.993^Age^ (x 1.018 if female) (x 1.159 if black); κ is 0.7 for females and 0.9 for males; ^α^ x is −0.329 for females and −0.411 for males; min indicates the minimum of serum creatinine (mg/dL)/κ or 1; max indicated the maximum of serum creatinine (mg/dL)/κ or 1. N-terminal pro brain natriuretic peptide (NT-proBNP) was measured by ElectroChemiLuminescence (ECL) on an Elecsys 2010 (Roche Diagnostics, Mannheim, Germany).

In course of the CAG the following measurements were obtained: left ventricular ejection fraction (LV-EF, *n* = 895), mean pulmonary artery pressure (mean PAP, *n* = 644), pulmonary capillary wedge pressure (PCWP, *n* = 626) and cardiac index (CI, *n* = 474).

### Classifications

CAD was classified into minor disease with a stenosis of 20–49%, one vessel disease (1VD), two vessel disease (2VD) and three vessel disease (3VD). ACS was scored in no ACS, unstable angina pectoris, non-ST elevation myocardial infarction (NSTEMI) and ST elevation myocardial infarction (STEMI). Angina pectoris complaints were classified according to the Canadian Cardiovascular Society (CCS) grading scale into Class I (ordinary physical activity does not cause angina), Class II (slight limitation of ordinary activity), Class III (marked limitation of ordinary physical activity) and Class IV (angina syndrome at rest) (31). Anemia was defined according to the criteria of the World Health Organization (WHO): hemoglobin <130 g/L in men and hemoglobin <120 g/L in women—severe anemia is defined as hemoglobin <80 g/L, moderate anemia is defined as hemoglobin 80−109 g/L and mild anemia is defined as hemoglobin 110–129 g/L in men and 110–119 g/L in women (32). We further subdivided anemic patients into those with anemia of chronic disease (ACD; TSAT <20% with ferritin >100 μg/L), iron deficiency anemia (IDA; TSAT <20% with ferritin <30 μg/L), ACD/IDA (TSAT <20% with ferritin 30–100 μg/L) or multifactorial anemia (TSAT >20%) (5). ID without anemia was defined as ferritin <100 μg/L plus TSAT <20% (absolute or true ID) or serum ferritin between 100–300 μg/L plus TSAT of <20% (functional ID) (33).

### Statistical Analysis

We used the Shapiro-Wilk test to test for Gaussian distribution. Parameters are depicted as *n* (%) or medians (25, 75th percentile), because they showed a skewed distribution. Mann-Whitney-*U* test, Kruskal-Wallis test or Pearson chi-square tests were used to test for significant differences among groups. Proportional hazard regression analysis was used to analyze the effects of risk factors on an adverse outcome. Multivariate proportional hazard regression analysis was stratified for sex and adjusted for established risk factors in patients with CAD including age, BMI, pack years, type 2 diabetes, hypertension, LDL, HDL, and triglyceride levels. Spearman rank correlation tests were performed to correlate continuous variables. All tests were two tailed and *p*-values < 0.05 were regarded as statistically significant. We performed Bonferroni correction to address type I errors in univariate analyses. Statistical analysis was performed using SPSS Statistics Version 26.0 for Macintosh (IBM Corporation, Armonk, NY, USA).

## Results

### Baseline Characteristics

We analyzed a data set comprising 2,082 patients (1,430 men and 652 women) in whom all preselected parameters were available with a median age of 63.4 years (56.2–70.6 years): 960 patients with chronic CAD (723 men and 237 women) and a median age of 64.5 years (58.0–71.1 years), 645 patients with ACS (475 men and 170 women) and a median age of 63.8 years (56.3–71.1 years), and 477 patients without CAD (232 men and 245 women) and a median age of 60.6 years (51.8–67.5 years; *p* < 0.001). The median follow-up was 9.81 years (8.58–10.53 years). By then 382 patients (18.4 %) died from cardio-cerebrovascular (CCV) disease and 218 patients (10.5%) from non-CCV diseases, while 12 patients were lost to follow-up. Baseline characteristics of patients with or without anemia are listed in Table 1.

**Table 1:** Baseline characteristics.

	**Total population**	**No anemia**	**Anemia**	**Significance**
	***n* = 2,082**	***n* = 1,725**	***n* = 357**	
	**Median (IQR) or %**	**Median (IQR) or %**	**Median (IQR) or %**	***p*-Value**
**Demographics and comorbidities**				
*Age [years]*	*63.4 (70.6–56.2)*	*62.6 (69.6–55.7)*	*68.3 (74.2–60.3)*	* <0.001*
Sex [men]	68.7%	69.5%	64.7%	0.075
*BMI [kg/m^2^]*	*27.1 (29.6–24.7)*	*27.2 (29.8–24.9)*	*26.1 (28.9–23.8)*	* <0.001*
Hypertension	52.0%	53.3%	45.4%	0.006
Atrial fibrillation	11.5%	11.3%	12.5%	0.495
*Diabetes mellitus Type 2*	*16.6%*	*15.2%*	*23.2%*	* <0.001*
Smoking	63.0%	62.6%	64.4%	0.741
Pack Years	9 (30–0)	9 (30–0)	8 (30–0)	0.527
*Lipid lowering therapy*	*49.8%*	*47.9%*	*58.5%*	* <0.001*
**Clincial characteristics**				
Heart rate [bpm]	68 (76–61)	68 (76–61)	68 (77–62)	0.111
*Syst. BP [mmHg]*	*140 (155–123)*	*140 (156–123)*	*136 (151–119)*	* <0.001*
*Diast. BP [mmHg]*	*80 (88–73)*	*81 (89–74)*	*75 (83–68)*	* <0.001*
CAD classification				* <0.001*
no CAD	*22.9%*	*25.3%*	*11.5%*	–
minor CAD	*10.7%*	*10.7%*	*10.9%*	–
one vessel disease	*19.5%*	*19.6%*	*19.3%*	–
two vessel disease	*18.5%*	*17.8%*	*22.1%*	–
three vessel disease	*28.3%*	*26.7%*	*36.1%*	–
CCS grading scale				* <0.001*
CCS class I	*38.8%*	*38.6%*	*39.5%*	–
CCS class II	*34.4%*	*36.3%*	*25.5%*	–
CCS class III	*15.5%*	*15.0%*	*18.2%*	–
CCS class IV	*11.3%*	*10.1%*	*16.8%*	–
NYHA class				0.006
NYHA class I	52.6%	54.0%	46.2%	–
NYHA class II	29.4%	29.2%	30.5%	–
NYHA class III/IV	18.0%	16.9%	23.2%	–
*LV-EF [%]*	*64 (73–47)*	*65 (74–49)*	*55 (70–37)*	* <0.001*
mean PAP [mmHg]	18 (25–15)	18 (24–15)	20 (30–15)	0.025
PCWP [mmHg]	10 (15–8)	10 (15–8)	12 (19–9)	0.021
CI [L/min/m^2^]	2.44 (3.00–2.10)	2.40 (3.00–2.10)	2.60 (3.10–2.10)	0.588
**Laboratory testing (serum)**				
*eGFR [mL/min/1.73 m^2^]*	*83.85 (96.54–70.14)*	*84.97 (97.51–72.00)*	*77.83 (89.92–59.92)*	* <0.001*
*Cholesterol [mg/dL]*	*206 (237–178)*	*211 (240–183)*	*182 (209–159)*	* <0.001*
*LDL [mg/dL]*	*116 (138–95)*	*119 (141–98)*	*104 (124–84)*	* <0.001*
*HDL [mg/dL]*	*37 (45–31)*	*38 (45–32)*	*34 (43–29)*	* <0.001*
*Triglycerides [mg/dL]*	*143 (198–107)*	*145 (202–110)*	*132 (174–98)*	* <0.001*
*hsCRP [mg/dL]*	*0.33 (0.83–0.13)*	*0.29 (0.69–0.12)*	*0.74 (2.08–0.26)*	* <0.001*
*Neopterin [nmol/L]*	*6.93 (8.56–5.67)*	*6.77 (8.28–5.57)*	*8.05 (11.55–6.57)*	* <0.001*
*Neopterin/eGFR ratio*	*0.083 (0.122–0.061)*	*0.079 (0.111–0.059)*	*0.106 (0.187–0.074)*	* <0.001*
*IL-6 [ng/L]*	*3.12 (6.08–1.74)*	*2.85 (5.34–1.67)*	*5.17 (10.09–2.61)*	* <0.001*
*IL-12 [ng/L]*	*65.33 (104.93–38.50)*	*63.19 (101.97–37.33)*	*75.70 (122.75–45.74)*	* <0.001*
*Fibrinogen [mg/dL]*	*376 (451–319)*	*368 (438–316)*	*431 (527–359)*	* <0.001*
*SAA [mg/L]*	*5.15 (12.40–2.90)*	*4.70 (9.50–2.80)*	*9.40 (39.90–3.80)*	* <0.001*
*TSAT [%]*	*25.22 (32.85–18.67)*	*26.41 (33.96–20.15)*	*18.74 (25.78–12.84)*	* <0.001*
Ferritin [ng/mL]	156 (274–89)	160 (276–91)	144 (256–78)	0.025
Hepcidin [ng/mL]	6.57 (10.16–4.03)	6.57 (9.87–4.11)	6.64 (11.83–3.28)	0.638
sTfR [mg/L]	1.26 (1.53–1.06)	1.25 (1.52–1.06)	1.26 (1.64–1.05)	0.435
Ferritin-index	0.581 (0.737–0.469)	0.579 (0.728–0.472)	0.593 (0.809–0.457)	0.192
*Hemoglobin [g/L]*	*13.90 (14.90–12.90)*	*14.20 (15.10–13.40)*	*11.80 (12.40–11.20)*	* <0.001*
Vitamin B12 [ng/L]	347 (473–259)	347 (467–264)	344 (515–237)	0.716
*Folic acid [μg/L]*	*8.00 (10.40–6.10)*	*8.20 (10.50–6.20)*	*7.40 (9.70–5.30)*	* <0.001*

*Parameters from 2,082 patients are listed as median (IQR) or n (%) for the total patients' cohort and for patients with or without anemia. Mann-Whitney U test and the Pearson chi-square test was used for comparisons between the two groups. Bonferroni correction was used to address type I errors for multiple testing thus p-Values <0.00128 were suggested as statistically significant*.

*BMI, body mass index; CAD, coronary artery disease; CCS, Canadian Cardiovascular Society; NYHA, New York Heart Association; Syst. BP, systolic blood pressure; Diast. BP, diastolic blood pressure; LV-EF, left ventricular ejection fraction; mean PAP, mean pulmonary artery pressure; PCWP, pulmonary capillary wedge pressure; CI, cardiac index**;** eGFR, estimated glomerular filtration rate; LDL, low density lipoprotein; HDL, high density lipoprotein; hsCRP, high-sensitive C-reactive protein; IL-6, interleukin 6; IL-12, interleukin 12; SAA, serum amyloid A; TSAT, transferrin saturation; sTfR, soluble transferrin receptor*.

At least one inflammatory marker (CRP or neopterin) was elevated in 988 patients (47.5 %) and the prevalence of that finding was significantly higher in CAD and ACS patients as compared to controls [177 patients without CAD (37.1%), 402 patients with chronic CAD (41.9%), 409 patients with ACS (63.4%)]. Among the specific immune biomarkers, 196 patients (9.4%) had only elevated neopterin levels (≥8.7 nmol/L), 494 patient (23.7%) had only elevated hsCRP levels (≥0.5 mg/dL) and 298 patients (14.3%) had both, elevated neopterin and hsCRP levels.

### Anemia Is Frequently Encountered and Related to Disease Severity and Immune Activation

Anemia was found in 357 patients (17.1%): 41 patients without CAD (8.6%), 155 patients with chronic CAD (16.1%) and 161 patients with ACS (25.0%, *p* <0.001). Grading of anemic patients showed that most patients presented with a mild anemia (*n* = 288, 13.8%), while 68 patients (3.3%) had a moderate and only one patient a severe anemia. The prevalence of anemia tended to be higher in women than in men (19.3 vs. 16.2%, *p* = 0.075); yet, in patients under the age of 55 years significantly more women were anemic (19.4 vs. 9.6%, *p* = 0.006).

Anemia was related to a more progressed CAD, higher CCS and NYHA classes, a significantly higher mean PAP and PCWP, and a significantly lower systolic BP, diastolic BP, eGFR and LV-EF. Anemic patients also had significantly higher neopterin, hsCRP, IL-6, IL-12, fibrinogen and serum amyloid A (SAA) levels, as well as a higher neopterin/eGFR ratio compared to non-anemic patients independent of sex and age (Table 1). Accordingly, hemoglobin levels correlated with neopterin, hsCRP, IL-6, IL-12, fibrinogen and SAA levels as well as with the neopterin/eGFR ratio independent of sex and age in patients with chronic CAD and ACS (Table 2).

**Table 2:** Spearman-rank correlations with hemoglobin.

	**Total population**	**Men**	**Women**
	***n* = 2,082**	***n* = 1,430**	***n* = 652**
hsCRP [mg/dL]	−0.226*	−0.253*	−0.182*
Neopterin [nmol/L]	−0.164*	−0.175*	−0.136*
Neopterin/eGFR ratio	−0.195*	−0.176*	−0.132*
IL-6 [ng/L]	−0.176*	−0.232*	−0.159*
IL-12 [ng/L]	−0.140*	−0.070	−0.188*
Fibrinogen [mg/dL]	−0.164*	−0.209*	−0.143*
SAA [mg/L]	−0.233*	−0.217*	−0.136*
TSAT [%]	0.351*	0.357*	0.307*
Ferritin [ng/mL]	0.188*	0.074	0.034
Hepcidin [ng/mL]	0.051	−0.014	0.027
sTfR [mg/L]	0.084*	0.105*	0.069
Ferritin-index	−0.018	0.057	0.058
Vitamin B12 [ng/L]	0.031	0.078	0.020
Folic acid [μg/L]	0.047	0.120*	0.023

Spearman-rank correlation analyses of hemoglobin with different parameters from 2,082 patients are listed for the total patients' cohort and separated for sex. Bonferroni correction was used to address type I errors for multiple testing thus p-Values <0.00119 were suggested as statistically significant (*); hsCRP, high-sensitive C-reactive protein; IL-6, interleukin 6; IL-12, interleukin 12; SAA, serum amyloid A; TSAT, transferrin saturation; sTfR, soluble transferrin receptor.

**Figure 2:**
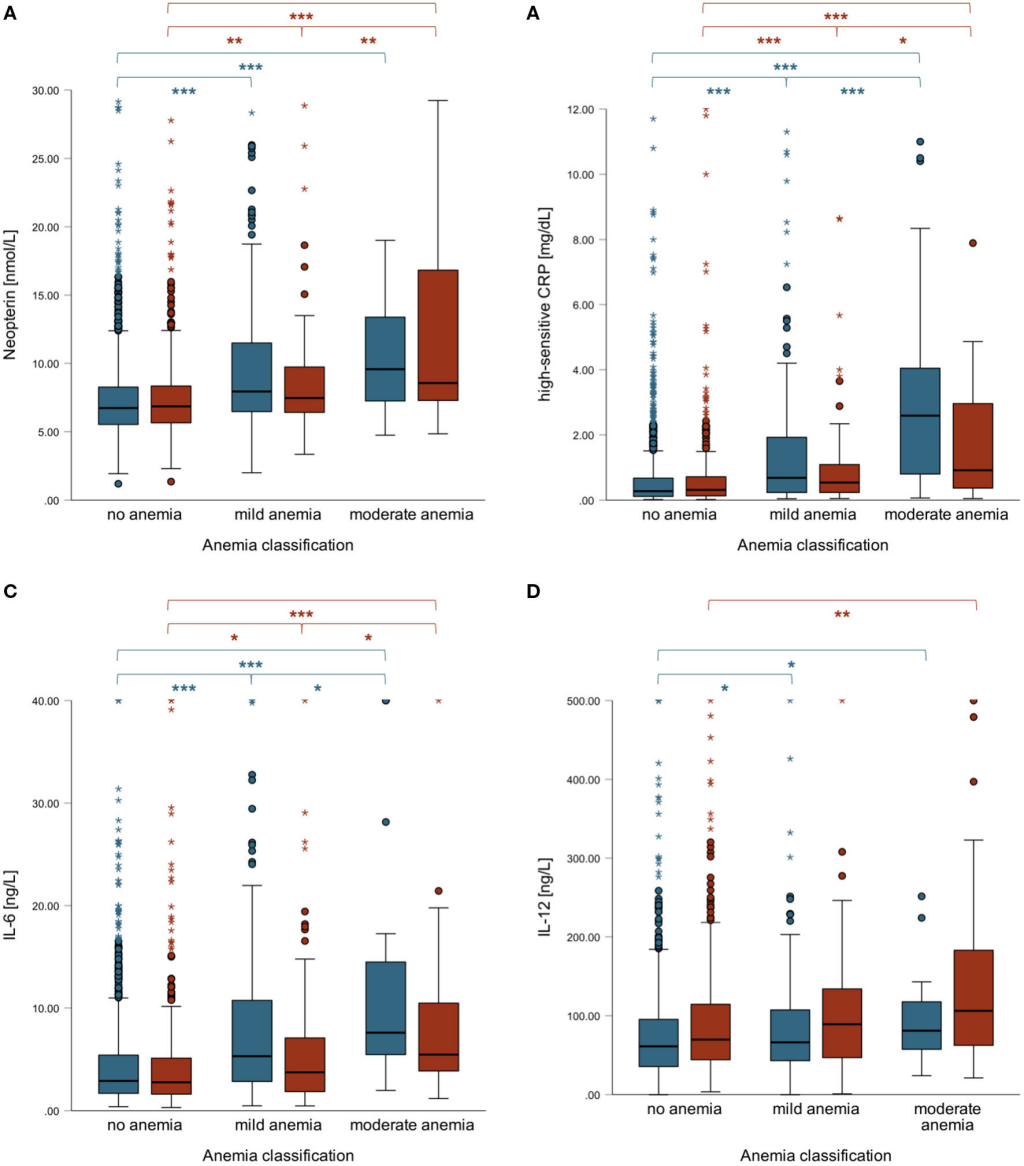
Boxplots of neopterin **(A)**, hsCRP **(B)**, IL-6 **(C)** and IL-12 levels **(D)** in men (blue) and women (red) according to anemia severity. Men with mild or moderate anemia had significantly higher IL-6 levels compared to women with mild (*p* = 0.007) or moderate anemia (*p* = 0.060). ***p < 0.001, **p < 0.01, *p < 0.05.

However, there were gender differences regarding inflammatory parameters and anemia: higher neopterin, hsCRP and IL-6 concentrations were positively related to anemia severity especially in men, while IL-12 rose with anemia severity especially in women (Figure 2). In fact, IL-12 levels were significantly higher in women compared to men in general (72.69 vs. 62.03 ng/L, *p* < 0.001) and were correlated with hemoglobin concentrations only in women (Table 2). Table 2 shows correlations of inflammatory parameters with hemoglobin concentrations for the total population and separately for men and women.

### Anemia Classification According to Iron Deficiency in Terms of Disease Severity and Immune Activation

We then studied patients according to the different types of anemia. Anemia of chronic disease (ACD) was present in 119 patients (5.7%; 84 men [5.9%] and 35 women [5.4%]), while 52 patients had ACD + IDA (2.5%; 29 men [2.0%] and 23 women [3.5%]). IDA was found in 28 patients (1.3%; 15 men [1.0%] and 13 women [2.0%]), and 158 patients had multifactorial or unclassified anemia (7.6%; 103 men [7.2%] and 55 women [8.4%]).

**Figure 3:**
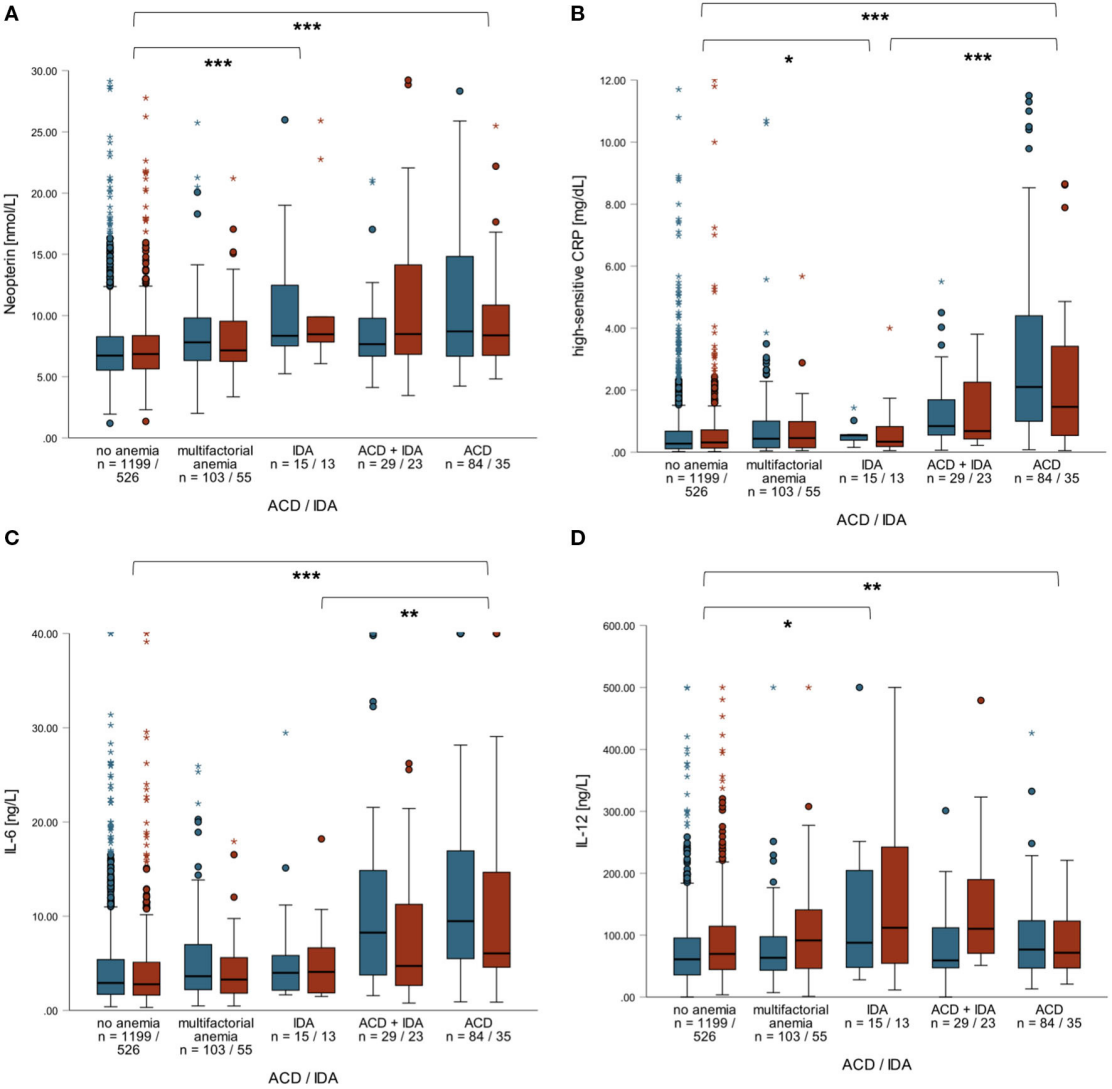
Boxplots of neopterin **(A)** hsCRP **(B)**, IL-6 **(C)** and IL-12 levels **(D)** in men (blue) and women (red) with or without ACD and/or IDA or multifactorial anemia. Neopterin and IL-12 levels did not significantly differ between patients with ACD, ACD+IDA or IDA independent of sex. Contrary, IL-6 and hsCRP levels were significantly higher in patients with ACD compared to patients with IDA or ACD + IDA (especially in men). ***p < 0.001, **p < 0.01, *p < 0.05.

Patients with ACD presented with a more progressed CAD and also with a higher CCS. Patients with ACD also differed from patients with IDA regarding laboratory parameters and also vital parameters (see Table 3). Patients with ACD (*n* = 119) had significantly higher neopterin, hsCRP, IL-6, IL-12, fibrinogen and SAA levels compared to non-anemic patients (*n* = 1,725). Interestingly, neopterin and IL-12 levels did not significantly differ among patients with ACD, ACD + IDA (*n* = 52) or IDA (*n* = 28; Figures 3A,B), while patients with ACD had significantly higher hsCRP, IL-6, fibrinogen and SAA levels compared to patients with IDA (Figures 3C,D). The latter finding was less pronounced in women compared to men.

**Table 3:** Baseline characteristics within the ACD/IDA classification.

	**no anemia**	**ACD**	**ACD + IDA**	**IDA**	**MfA**	**Sig**.
	***n* = 1,725**	***n* = 119**	***n* = 52**	***n* = 28**	***n* = 158**	
	**Median or %**	**Median or %**	**Median or %**	**Median or %**	**Median or %**	***p*-Value**
**Demographics and comorbidities**						
*Age [years]*	*62.63*	*68.14*	*70.90*	*69.04*	*67.52*	* <0.001*
Sex [men]	69.5%	70.6%	55.8%	53.6%	65.2%	0.071
*BMI [kg/m^2^]*	*27.18*	*26.10*	*26.00*	*26.71*	*26.09*	* <0.001*
*Hypertension*	*53.3%*	*34.5%*	*51.9%*	*67.9%*	*37.5%*	* <0.001*
Atrial fibrillation	11.3%	11.9%	18.4%	14.3%	10.9%	0.617
*Diabetes mellitus Type 2*	*15.2%*	*24.4%*	*25.0%*	*25.0%*	*21.5%*	*0.006*
Smoking	62.6%	68.9%	53.8%	57.2%	65.8%	0.311
Pack Years	9.0	12.5	0.9	1.2	10.0	0.171
*Lipid lowering therapy*	*47.9%*	*65.5%*	*55.8%*	*50.0%*	*55.7%*	*0.002*
**Clinical characteristics**						
*Heart rate [bpm]*	*67.67*	*71.00*	*67.67*	*70.67*	*66.00*	*0.013*
*Syst. BP [mmHg]*	*140.00*	*129.00*	*141.50*	*145.00*	*137.33*	* <0.001*
*Diast. BP [mmHg]*	*81.33*	*71.33*	*74.33*	*80.00*	*76.33*	* <0.001*
CAD classification						* <0.001*
no CAD	*25.3%*	*8.4%*	*9.6%*	*17.9%*	*13.3%*	–
minor CAD	*10.7%*	*6.7%*	*7.7%*	*14.3%*	*14.6%*	–
one vessel disease	*19.6%*	*17.6%*	*23.1%*	*21.4%*	*19.0%*	–
two vessel disease	*17.8%*	*28.6%*	*15.4%*	*10.7%*	*21.5%*	–
three vessel disease	*26.7%*	*38.7%*	*44.2%*	*35.7%*	*31.6%*	–
CCS grading scale						* <0.001*
CCS class I	*38.6%*	*42.0%*	*34.6%*	*50.0%*	*37.3%*	–
CCS class II	*36.3%*	*18.5%*	*26.9%*	*21.4%*	*31.0%*	–
CCS class III	*15.0%*	*16.8%*	*21.2%*	*21.5%*	*17.7%*	–
CCS class IV	*10.1%*	*22.7%*	*17.3%*	*7.1%*	*13.9%*	–
*NYHA class*						*0.003*
*NYHA class I*	*54.0%*	*52.1%*	*36.5%*	*39.3%*	*46.2%*	–
*NYHA class II*	*29.2%*	*26.1%*	*36.5%*	*17.9%*	*34.2%*	–
*NYHA class III/IV*	*16.9%*	*21.8%*	*26.9%*	*42.9%*	*19.6%*	–
*LV-EF [%]*	*65*	*42*	*46*	*64*	*60*	* <0.001*
mean PAP [mmHg]	18	21	21	26	19	0.084
PCWP [mmHg]	10	13	12	15	11	0.058
CI [L/min/m^2^]	2.40	2.60	2.65	3.00	2.30	0.527
**Laboratory testing (serum)**						
*eGFR [mL/min/1.73 m^2^]*	*84.97*	*77.83*	*73.90*	*74.15*	*80.81*	* <0.001*
*Cholesterol [mg/dL]*	*211*	*177*	*180*	*200*	*186*	* <0.001*
*LDL [mg/dL]*	*119*	*101*	*102*	*109*	*105*	* <0.001*
*HDL [mg/dL]*	*38*	*31*	*36*	*43*	*36*	* <0.001*
*Triglycerides [mg/dL]*	*145*	*138*	*119*	*119*	*134*	*0.001*
*hsCRP [mg/dL]*	*0.29*	*1.93*	*0.79*	*0.52*	*0.43*	* <0.001*
*Neopterin [nmol/L]*	*6.77*	*8.40*	*8.05*	*8.40*	*7.42*	* <0.001*
*Neopterin/eGFR ratio*	*0.079*	*0.118*	*0.110*	*0.115*	*0.093*	* <0.001*
*IL-6 [ng/L]*	*2.85*	*8.28*	*6.42*	*4.06*	*3.41*	* <0.001*
*IL-12 [ng/L]*	*63.19*	*74.76*	*81.40*	*89.71*	*69.41*	*0.001*
*Fibrinogen [mg/dL]*	*368*	*514*	*432*	*387*	*393*	* <0.001*
*SAA [mg/L]*	*4.70*	*35.00*	*9.70*	*6.15*	*5.70*	* <0.001*
*TSAT [%]*	*26.41*	*13.53*	*14.97*	*10.49*	*26.70*	* <0.001*
*Ferritin [ng/mL]*	*160*	*196*	*65*	*15*	*167*	* <0.001*
*Hepcidin [ng/mL]*	*6.57*	*9.10*	*4.62*	*0.49*	*7.28*	* <0.001*
*sTfR [mg/L]*	*1.25*	*1.30*	*1.41*	*2.10*	*1.17*	* <0.001*
*sTfR-ferritin-index*	*0.579*	*0.531*	*0.773*	*1.869*	*0.530*	* <0.001*
*Hemoglobin [g/L]*	*14.20*	*11.60*	*11.80*	*11.50*	*11.90*	* <0.001*
Vitamin B12 [ng/L]	347	339	357	363	341	0.896
*Folic acid [μg/L]*	*8.20*	*6.80*	*7.55*	*8.45*	*7.65*	* <0.001*

*Parameters from 2,082 patients are listed as median (IQR) or n (%) for patients within the ACD/IDA classification. Kruskal-Wallis test and the Pearson chi-square test was used for comparisons between all subgroups. Bonferroni correction was used to address type I errors for multiple testing thus p-Values <0.00128 were suggested as statistically significant*.

*MfA, multifactorial anemia; IDA, iron deficiency anemia; ACD, anemia of chronic disease; Sig., significance; BMI, body mass index; CAD, coronary artery disease; CCS, Canadian Cardiovascular Society; NYHA, New York Heart Association; Syst. BP, systolic blood pressure; Diast. BP, diastolic blood pressure; LV-EF, left ventricular ejection fraction; mean PAP, mean pulmonary artery pressure; PCWP, pulmonary capillary wedge pressure; CI, cardiac index; eGFR, estimated glomerular filtration rate; LDL, low density lipoprotein; HDL, high density lipoprotein; hsCRP, high-sensitive C-reactive protein; IL-6, interleukin 6; IL-12, interleukin 12; SAA, serum amyloid A; TSAT, transferrin saturation; sTfR, soluble transferrin receptor*.

Most of the patients with multifactorial anemia had a normocytic, normochromic anemia (*n* = 136, 86.1%) and presented with elevated neopterin and/or hsCRP levels (*n* = 96, 60.8%), which are hallmarks of ACD.

### Impact of Anemia and Immune Activation on Cardio-Cerebrovascular Mortality

**Figure 4:**
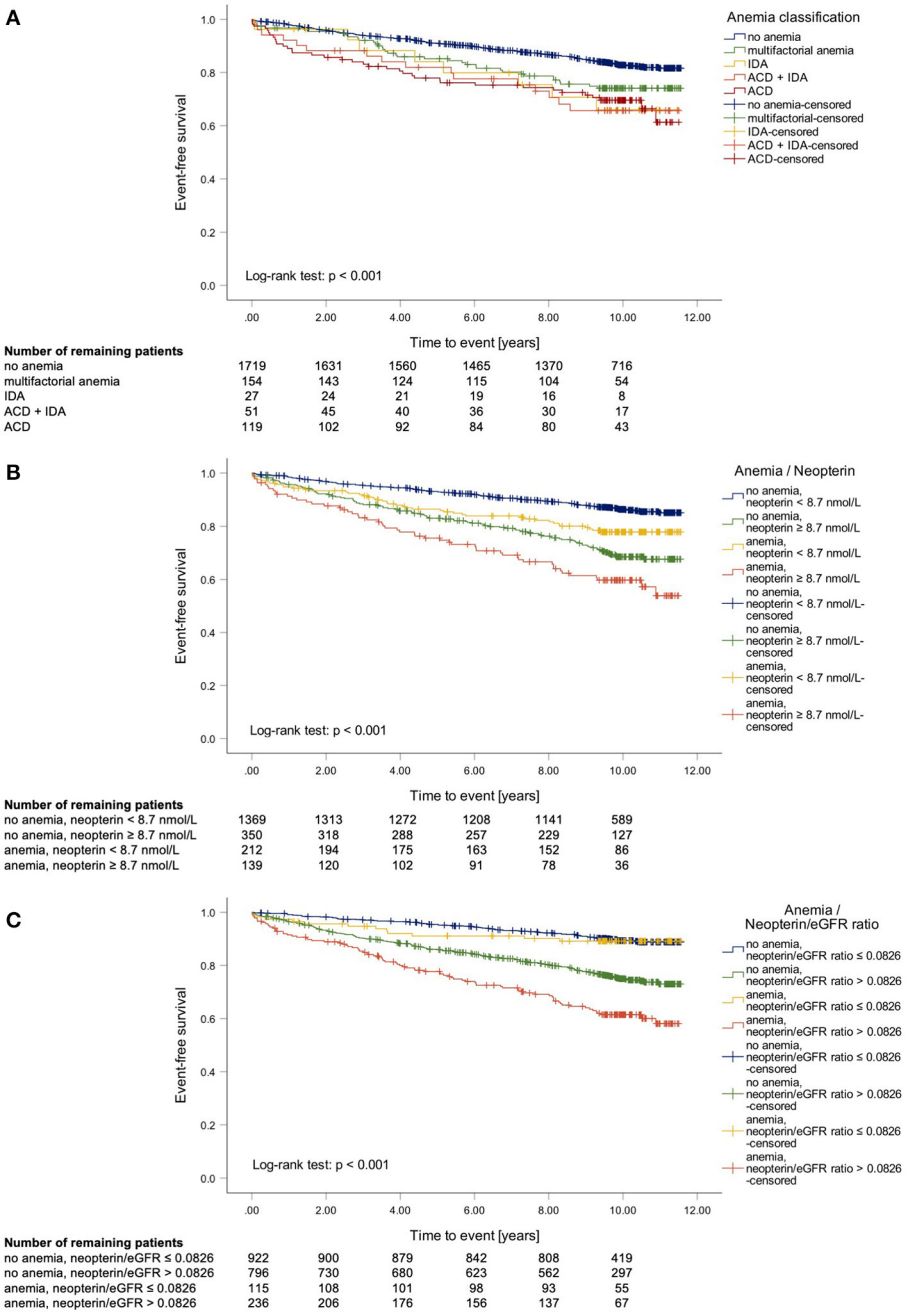
Kaplan-Meier curves of cardio-cerebrovascular (CCV) mortality in terms of the anemia subdivision into anemia of chronic disease (ACD) and iron deficiency anemia (IDA) **(A)** and Kaplan-Meier curve of CCV mortality in terms of anemia and elevated neopterin levels [≥8.7 nmol/L; **(B)**] or respectively neopterin/eGFR ratio [>0.0826; **(C)**].

In univariate Cox regression analysis, the presence of anemia was linked to an almost 2-fold higher CCV mortality (HR1.914 [95%CI 1.521–2.408]). In addition, multivariate Cox regression analysis stratified for sex and adjusted for established risk factors in patients with CAD including age, BMI, LDL, HDL, triglycerides, pack years, hypertension, or type 2 diabetes confirmed that the presence of anemia was linked to a significantly higher CCV mortality (HR 1.348 [95%CI 1.052–1.727]). Further classification of anemic patients showed that those with ACD (with and without concomitant IDA) had the highest CCV event rate within the following 2 years (ACD 14.3%; ACD + IDA 11.4%; IDA 3.6%; multifactorial anemia 4.5%; no anemia 4.0%). Interestingly, after 10 years of follow-up mortality rates did not differ between patients with ACD and anemia of other cause (Figure 4A).

In patients with multifactorial anemia those with elevated inflammatory markers also had a significantly higher CCV mortality (HR 2.095 [95%CI 1.014–4.332]). Especially, patients with elevated neopterin concentrations (HR 2.137 [95%CI 1.120–4.079]) were at a higher risk to die by a CCV event.

Finally, we studied the impact of inflammation on the outcome of CAD patients and investigated whether inflammatory markers were linked to the presence of anemia. Cox-regression analysis revealed a significant interaction of neopterin and hemoglobin (neopterin x hemoglobin: *p* = 0.031; neopterin/eGFR ratio x hemoglobin: *p* = 0.024). Accordingly, Kaplan-Meier curves show that patients with elevated neopterin levels (or an elevated neopterin/eGFR ratio to account also for renal function) had a significantly higher CCV mortality rate independent of concomitant anemia. However, anemia further increased the risk for subsequent events in patients with elevated neopterin levels (HR 1.487 [95%CI 1.069–2.069], Figure 4B) or with an elevated neopterin/eGFR ratio (HR 1.737 [95%CI 1.346–2.241], Figure 4C). Interestingly, in patients with a low neopterin/eGFR ratio (defined by the median), anemia was not related to a significantly higher CCV event rate Figure 4C).

## Discussion

Our findings confirm that anemia is related to an adverse outcome of patients with cardiovascular disease (34, 35). We could show for the first time that the underlying cause of anemia also has a significant impact on the prognosis of patients: ACD, which is caused by immune activation, is associated with a significantly higher risk for cardio-cerebrovascular events in the subsequent 2 years when compared to patients with IDA or multifactorial anemia. Our data further indicate that inflammation is the underlying cause for the development of anemia in a high percentage of patients with CAD. Therefore, it might be useful to differentiate between patients with ACD, IDA or multifactorial anemia to better predict their risks to die within the next years and to choose the best therapy option.

Anemia was linked to CAD severity and predictive for all-cause mortality—similar to elevated neopterin levels. However, anemia was only predictive for an adverse outcome in patients with elevated inflammatory markers (and a higher neopterin/eGFR ratio, respectively). Thus, our data suggest that anemia reflects disease severity and predicts outcome mainly because it is strongly associated with inflammation. Since also decreasing renal function during aging can contribute to the development of anemia, we also accounted for this point by calculating the neopterin/eGFR ratio. Recently, advanced inflammation and reduced kidney function were shown to contribute to a poor clinical course in patients with chronic heart failure (35).

In line with this hypothesis, patients with ACD also had a poorer prognosis than subjects with multifactorial anemia—pointing to the crucial relationship between anemia, iron homeostasis and inflammation in subjects with cardiovascular diseases (36). In our study population, more than 80% of patients with multifactorial anemia had a normocytic, normochromic anemia and presented with elevated inflammatory markers which are both typical for ACD. However, according to established classifications (9, 33), those patients were not classified as having ACD because TSAT was above 20%. Still, the finding that ferritin levels were similar to ACD subjects in patients with multifactorial anemia indicates that chronic inflammation might also contribute importantly to the development of anemia in these patients. Also, the fact that patients with elevated inflammatory markers had a higher CCV risk in this subgroup of patients suggests that the inflammatory process may underlie CAD progression as well as the development of anemia. Higher TSAT in patients with multifactorial anemia may also result from low transferrin levels as a consequence of inflammation (thereby masking the ACD phenotype) (37).

The hypothesis that inflammation contributes to the development of anemia in patients with cardiovascular disease, is also supported by a recently published *post hoc* analysis of the CANTOS trial (38). In patients with previous myocardial infarction rising hsCRP concentrations were related to an increasing incidence rate of anemia. In patients receiving canakinumab—an antibody that is targeting IL-1β—hsCRP and IL-6 levels decreased while hemoglobin levels increased concomitantly in patients with baseline anemia (38). Furthermore, a reduced incidence of anemia could be demonstrated in patients treated with canakinumab compared to patients receiving placebo (38). This indicates that anti-inflammatory strategies can improve anemia most likely by ameliorating inflammation-driven disturbances of iron homeostasis and cytokine mediated effects on erythropoiesis (37).

Unfortunately, the CANTOS trial did not investigate different anemia types, which might in fact provide interesting new results. Still, also other data support our hypothesis, that inflammation is causally involved in CAD progression and anemia development: Anti-inflammatory treatment with colchicine reduces the secondary attack rate in CAD (39), and ameliorates anemia in CAD as shown in Familial Mediterranean fever (40).

In our population, patients with ACD had significantly higher hsCRP, IL6, fibrinogen and SAA levels when compared to patients with IDA or multifactorial anemia. Furthermore, ACD patients had the most advanced disease progression and the poorest short-time outcome. ACD is characterized by iron restriction by macrophages (5), while IDA is characterized by decreased tissue iron stores (41). Both are resulting in decreased erythropoiesis over time due to reduced iron availability (5, 41). Iron was shown earlier to reduce the efficiency of the IFN-γ signal in monocytes, thus also decreasing neopterin production (42) and cellular Th1 immune response (43). Therefore, in patients with IDA, low iron availability may enhance Th1 immune response.

Finally, we could also show some significant differences in immune activation between anemic men and women. Anemic men (especially male patients with ACD) had higher neopterin, hsCRP and IL-6 levels compared to anemic women, while anemic women had higher IL-12 levels compared to anemic men. The above-mentioned mechanisms of inflammation affecting erythropoiesis might not be as distinctive in women as in men. Since women have a higher prevalence of anemia especially before menopause because of their menstrual bleeding, they might have some sort of protection mechanism preventing an additional decrease of erythropoiesis due to inflammation. Actually, IL-12 was shown to enhance erythropoiesis *in vitro* (44), suggesting that higher IL-12 levels observed in women may counteract the inflammatory burden on erythropoiesis.

### Limitations

Neopterin levels were not available for all patients that were initially included in the study, which is why the findings made in this study do not allow unrestricted generalization to CAD patients in general. Also, the fact that data concerning concomitant erythropoietin therapy or blood transfusion were not available is a limitation of this study. Unfortunately, we do not have follow-up data regarding anemia and inflammation of patients—these data would certainly provide very interesting information.

## Conclusion

This study confirms that anemia is common and strongly related to immune activation in patients with coronary artery disease. Specifically, the combination of anemia and inflammation is associated with a worse prognosis and an increased risk of cardio-cerebrovascular death in patients with CAD. Actually, anemia was only predictive for further cardio-cerebrovascular events in patients with elevated inflammatory markers. We could also show differences in immune activation between anemic men and women.

In summary, our data suggest that the association of anemia with disease severity and outcome might mainly be due to underlying inflammation; additionally, advanced renal dysfunction should also be taken into account.

## Data Availability Statement

The raw data supporting the conclusions of this article will be made available by the authors, without undue reservation.

## Ethics Statement

The studies involving human participants were reviewed and approved by Ethics committee at the Landesärztekammer Rheinland-Pfalz. The patients/participants provided their written informed consent to participate in this study.

## Author Contributions

WM: conceptualization and project administration. DF, TG, KK, WM, and GW: methodology. LL: software, formal analysis, and visualization. KK, LL, and GW: validation. DF, TG, MK, WM, and HS: investigation, resources, and data curation. KK and LL: writing—original draft preparation. DF, TG, MK, WM, HS, and GW: writing—review and editing. KK: supervision. All authors have read and agreed to the published version of the manuscript.

## Conflict of Interest

WM reports employment with Synlab Holding Deutschland GmbH, during the conduct of the study; grants from Siemens Healthineers, grants and personal fees from Aegerion Pharmaceuticals, grants and personal fees from AMGEN, grants from Astrazeneca, grants and personal fees from Sanofi, grants and personal fees from Amryt Pharmaceuticals, grants and personal fees from BASF, grants and personal fees from Abbott Diagnostics, grants and personal fees from Numares AG, grants and personal fees from Berlin-Chemie, grants and personal fees from Akzea Therapeutics, grants from Bayer Vital GmbH, grants from bestbion dx GmbH, grants from Boehringer Ingelheim Pharma GmbH Co KG, grants from Immundiagnostik GmbH, grants from Merck Chemicals GmbH, grants from Novartis Pharma GmbH, grants from Olink Proteomics, grants and personal fees from AMGEN, personal fees from Novartis Pharma, personal fees from Vifor Pharma, all outside the submitted work. The remaining authors declare that the research was conducted in the absence of any commercial or financial relationships that could be construed as a potential conflict of interest.

## Publisher's Note

All claims expressed in this article are solely those of the authors and do not necessarily represent those of their affiliated organizations, or those of the publisher, the editors and the reviewers. Any product that may be evaluated in this article, or claim that may be made by its manufacturer, is not guaranteed or endorsed by the publisher.
